# The Size of Human Mesenchymal Tissue Stem Cell Turnover Units is a Major Determinant for Maintaining High Stem Cell Fractions With Serial Culture Expansion

**DOI:** 10.1155/sci/1216876

**Published:** 2026-01-16

**Authors:** James L. Sherley, Chen Cao, Celia Sommer, Alex Dahlkemper, James Sugai, Hitesh Chopra, Darnell Kaigler

**Affiliations:** ^1^ Asymmetrex LLC, Boston, Massachusetts, USA; ^2^ Department of Periodontics and Oral Medicine, School of Dentistry, University of Michigan, Ann Arbor, Michigan, USA, umich.edu; ^3^ Department of Biomedical Engineering, University of Michigan, Ann Arbor, Michigan, USA, umich.edu

**Keywords:** alveolar bone, asymmetric self-renewal, cell lineage, expansion culture, mesenchymal stem cells, tissue stem cells, turnover unit

## Abstract

Recently, we used kinetic stem cell (KSC) counting to show that the stability of the stem cell fraction (SCF) during serial culture expansion of human oral alveolar bone mesenchymal tissue cell (MTC) preparations varied significantly among patient donors. Whereas some patient donor samples’ SCFs declined rapidly, others showed a moderate decrease; and still others had highly stable SCFs during serial culture expansion. Defining the cell kinetics basis for these differences in SCF stability could lead to effective solutions for the problem of loss of mesenchymal stem cell (MSC) function when MTC preparations are expanded for research and clinical applications. Using KSC counting, we show that greater SCF stability is associated with smaller stem cell turnover units, which reduce the SCF by cell dilution. This finding confirms earlier evidence and proposals that, by limiting the production and proliferation of committed progenitor cells, more effective expansion of tissue stem cells, such as MSCs, can be achieved. Because of the universal nature of tissue stem cell turnover units and the continuation of their basic cell kinetics programs in in vitro cell culture, the effects defined herein are predicted to apply to other types of human tissue stem cells of interest for stem cell medicine.

**Summary**

• Computational simulation can be used to define the stem cell‐specific fraction of tissue cell samples and its stability during cell expansion culture.
• The stem cell‐specific fraction often declines significantly during cell expansion culture.
• Declines in the stem cell‐specific fraction during expansion cell culture are attributable to the dilution of stem cells among the progeny nonstem cells that stem cells’ own special multiplication produces.

## 1. Introduction

Mesenchymal tissue cell (MTC) preparations are a leading focus for the development of new cellular therapies for both human medicine [1–4] and veterinary medicine [5, 6]. Only hematopoietic cell preparations command a comparable degree of medical interest and clinical trials activity. Donor tissue sources for MTCs include bone marrow, umbilical cord and other perinatal tissues, adipose tissue, and other mesenchymal cell‐containing tissues.

Because of the widely recognized cell heterogeneity of MTC preparations, recommendations have been made that such tissue cell populations be called “mesenchymal stromal cells” instead of their originating name, “mesenchymal stem cells” (MSCs) [7–9]. This change in name corrects an inaccuracy in the characterization of MTC preparations. Their high degree of cell subtype heterogeneity makes it unlikely that these preparations ever contain only MSCs. Lineage‐committed progenitor cells and differentiated cells are also present in MTC isolations; and nonstem cell subtypes continue to be produced when isolated MTC preparations are cultured with the intent of expanding MSCs in biomanufacturing processes [6, 10–12].

Though the revised name “stromal” avoids misrepresenting MTC preparation as containing only MSCs, its use has led to another problem. The term “mesenchymal stromal cells” is often applied as a license to ignore the unquantified MSC fraction of MTC samples altogether [7]. In either case, whether MTC preparations and production samples are called “stem” cells or “stromal” cells, the more important issue faced by the mesenchymal cell therapy field, academic and industry, is that both usages are often accompanied by overlooking the shortcomings of MTC preparations, production samples, and treatment samples whose cell heterogeneity is unquantified. Changes in the fractions of stem cells, progenitor cells, and differentiated cells that occur during biomanufacturing of MTCs are predicted to be critical quality attributes (CQAs) for patient treatment outcomes [12].

Kinetic stem cell (KSC) counting is a new method for determining the specific fractions of stem cells, progenitor cells, and arrested cells in tissue cell preparations and for determining how they change during serial cell culture [11–13]. In a recently published report [14], we used KSC counting to quantify the cell subtype heterogeneity of alveolar bone‐derived MTC preparations from eight dental patients. In addition to establishing significant inter‐donor variation in the initial stem cell fraction (SCF) of patient MTC preparations, we also noted significant variance in the stability of the SCF of the samples during serial culture expansion. The patient samples could be grouped into three distinct SCF stability categories: low stability, moderate stability, or stable, meaning no significant decrease in the SCF with serial culture [14].

Though the loss of stem cell function during expansion culture of MTCs is a well‐described challenge in MSC biomanufacturing [1, 15–22], KSC counting analyses were the first studies to show that it can be attributed at least in part to a decline in the SCF of MTC cultures [11, 12]. Understanding the cellular basis for such declines in mesenchymal SCF would enable the development of better biomanufacturing processes for MSC expansion for use in research and stem cell medicine. In the present report, we describe the use of KSC counting to provide evidence that a specific cell kinetics factor, the stem cell turnover unit size, is a major determinant of the stability of the SCF during MTC serial cell culture. From this new insight, it follows that strategies to reduce the MSC turnover unit size during cell culture will promote MSC expansion. KSC counting can be used to develop and optimize such innovation.

## 2. Materials and Methods

### 2.1. Oral Alveolar Bone Tissue Cell Derivation

In a previous study [14], the MTC populations used to obtain the analysis data for this report were derived from alveolar bone samples isolated in accordance with the University of Michigan School of Dentistry Institutional Review Board under approved protocol aBMSCs, IRB# #HUM00034368. The alveolar bone specimens were obtained from patients undergoing routine oral surgical procedures as previously described [14, 23]. Written informed consent was obtained from the subjects, who donated the cells for use in the study, at the School of Dentistry, University of Michigan.

### 2.2. TORTOISE Test Computational Simulation Analyses

Mean initial cell kinetics factor data (Figure 1) were derived as previously described using the KSC counting TORTOISE Test computational simulation software [11–13]. The turnover division number (TDN), is an output of TORTOISE Test software analysis. The serial culture mean cumulative population doubling (CPD) and mean dead cell fraction (DCF) data input for the analyses have been described [14] and are posted in a public database for open access [24]. The mean initial cell kinetics factor values were derived from 10 independent TORTOISE Test computer simulations as described previously [12, 13]. Cell subtype‐specific percentages during serial cell culture were computed from single‐example CPD data computational simulations that had fRMSE determinations ≤0.15 (Figures 2 and 3). The fRMSE = root mean squared error/the experimental CPD maximum. The fRMSE value indicates the quality of an evaluated TORTOISE Test CPD data simulations’ approximation of the respective experimental mean CPD data. The fRMSE is a different metric than the simulation quality score (SQS) in Figure 1 [11–13].

Figure 1Initial mean cell kinetics factors defined by KSC counting analyses of human alveolar bone‐derived MTC samples. Each diagram depicts the underlying KSC counting cell kinetics model with the respective initial mean cell kinetics factors determined for each of the 8 patient donor MTC preparations analyzed. (A, B) Patients 1 and 4, respectively; low SCF stability. (C–E) Patients 3, 8, and 5, respectively; moderate SCF stability. (F–H) Patients 2, 6, and 7, respectively; stable SCF. Symmetric stem cell divisions (left) and asymmetric stem cell divisions (right) can be distinguished by their different cell products. Bivalent circles, stem cells; uniform circles, transiently‐dividing committed progenitor cells; squares, terminally‐arrested cells; amorphous shapes, dead cells. SQS, mean simulation quality score. (#‐#), mean SQS 95% confidence interval. %, mean percent of cells of the labeled type at the start of serial culture. (#‐#%), 95% confidence intervals about the mean % values. RS, the rate of symmetric divisions by stem cells. NS, not significantly different than 0.0. SCF, initial mean MSC fraction. h, indicated cell cycle time in hours. TDN, mean turnover division number. (# cells), number of cells in a complete stem cell turnover unit.  ^∗^, the dead cell fraction (DCF) of terminally arrested cells is not a KSC counting software output. It is an input based on the measured mean total DCF across the entire period of serial cell culture [11].

**Figure:**
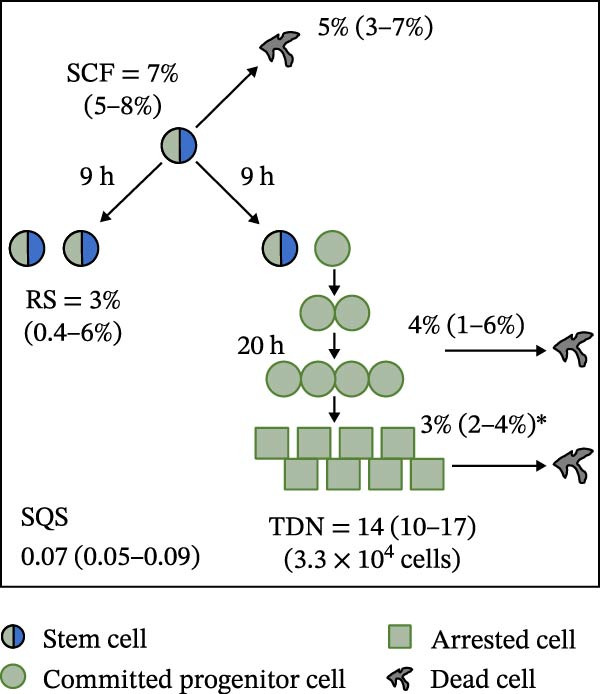
(A)

**Figure:**
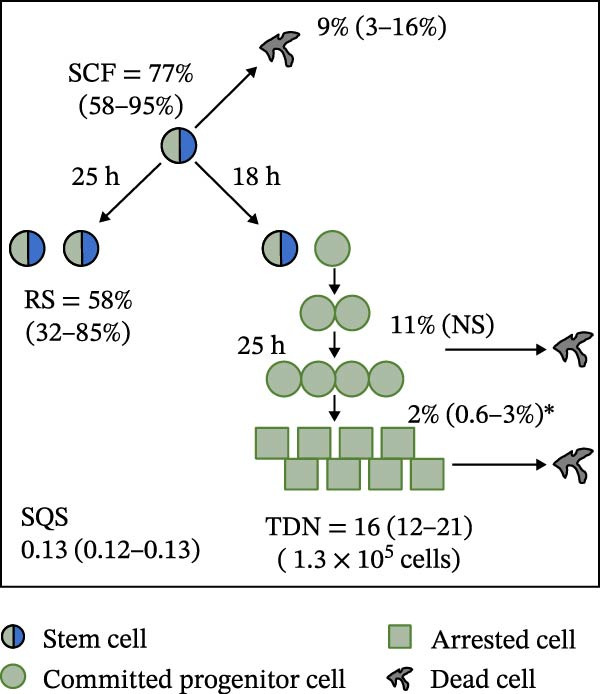
(B)

**Figure:**
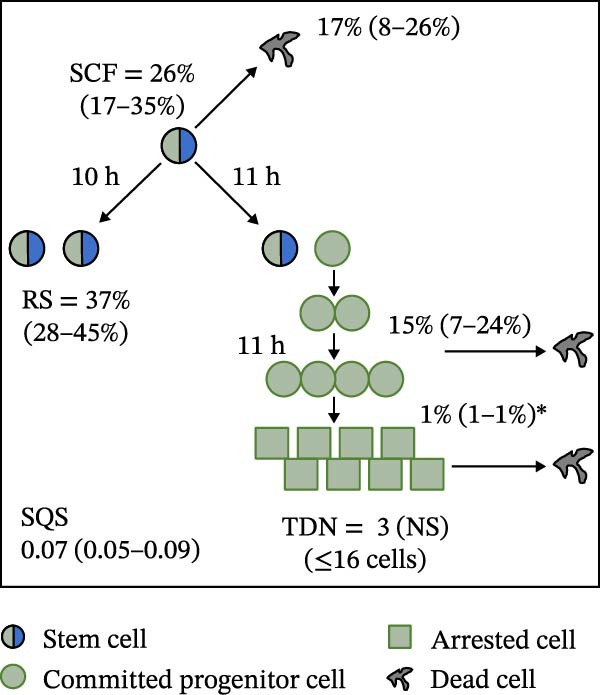
(C)

**Figure:**
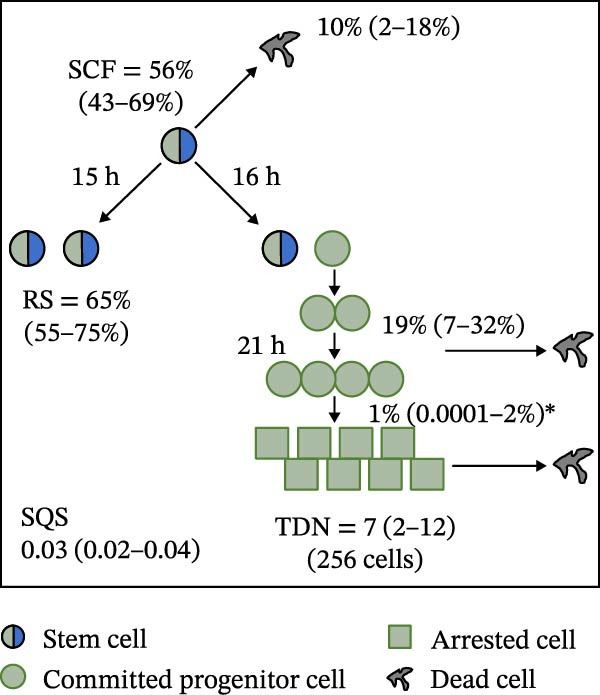
(D)

**Figure:**
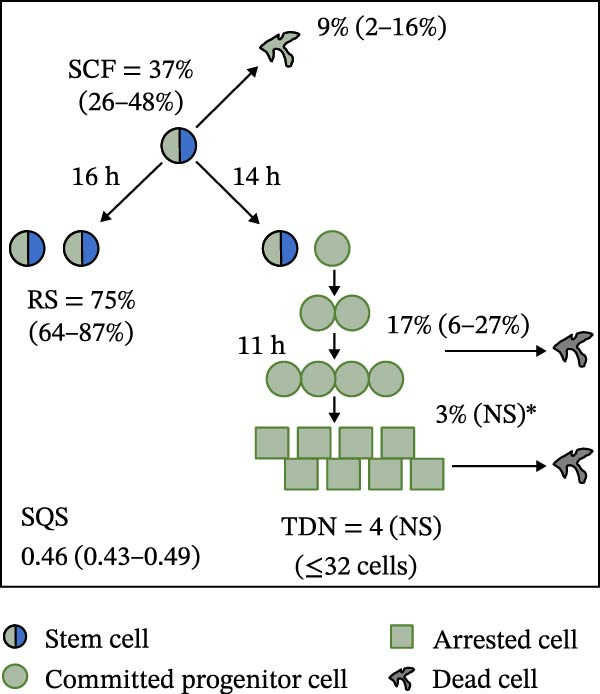
(E)

**Figure:**
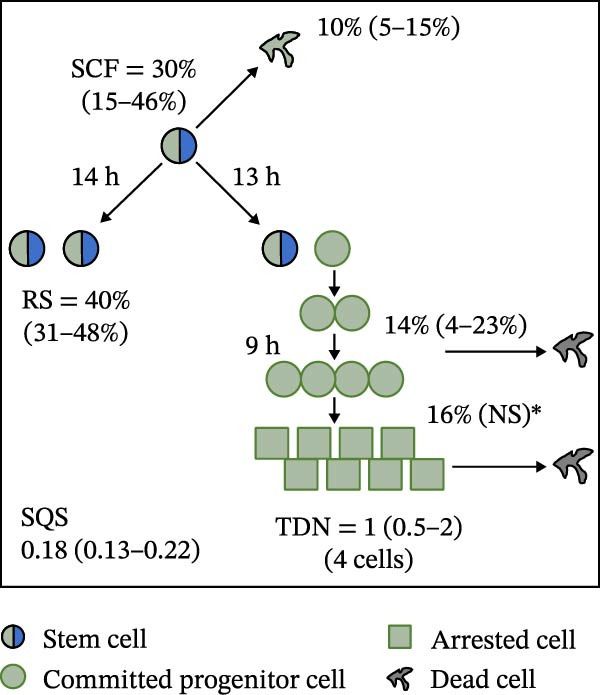
(F)

**Figure:**
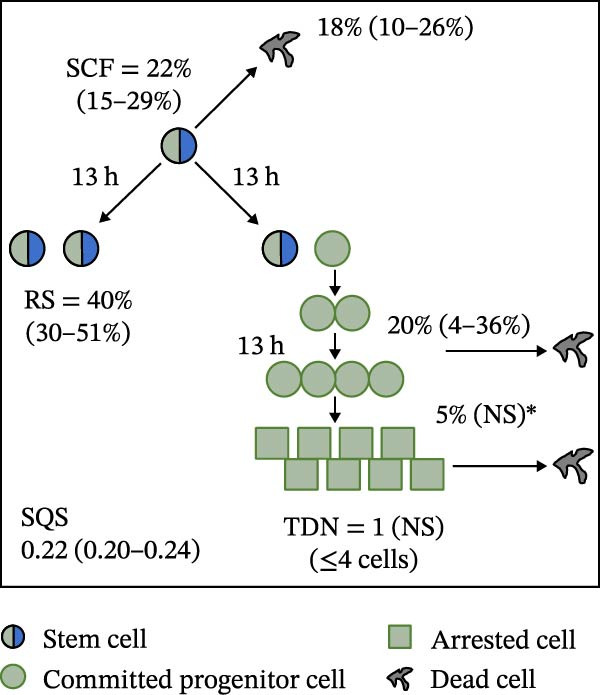
(G)

**Figure:**
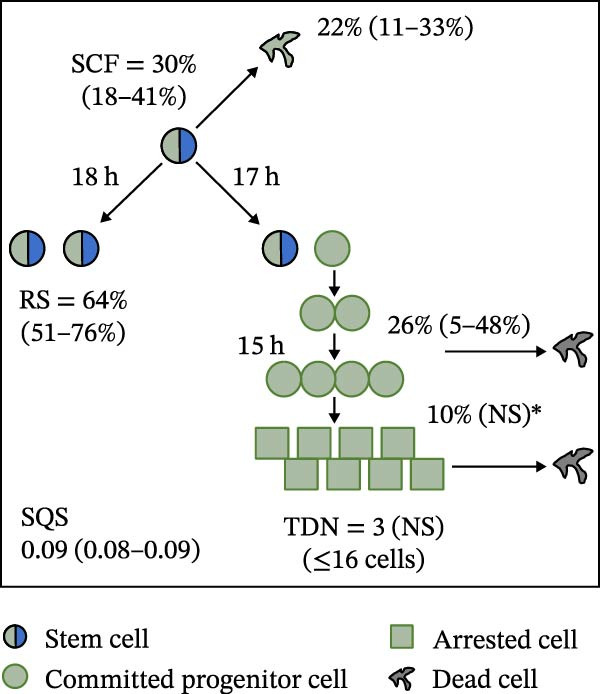
(H)

Figure 2KSC counting TORTOISE Test computational simulations of experimental mean CPD data for human alveolar bone‐derived MTC samples. Shown are the computational simulations of the mean CPD data that correspond to the respective cell subtype‐specific serial culture cell kinetics profiles shown in Figure 3. (A, B) Patients 1 and 4, respectively; low SCF stability. (C–E) Patients 3, 8, and 5, respectively; moderate SCF stability. (F–H) Patients 2, 6, and 7, respectively; stable SCF. Black lines, previously reported experimental mean CPD data for samples [14, 24]. Blue lines, instant simulated mean CPD data. fRMSE, the fractional root mean squared error/maximum mean CPD value, for the instant simulated data compared to the experimental CPD data.

**Figure:**
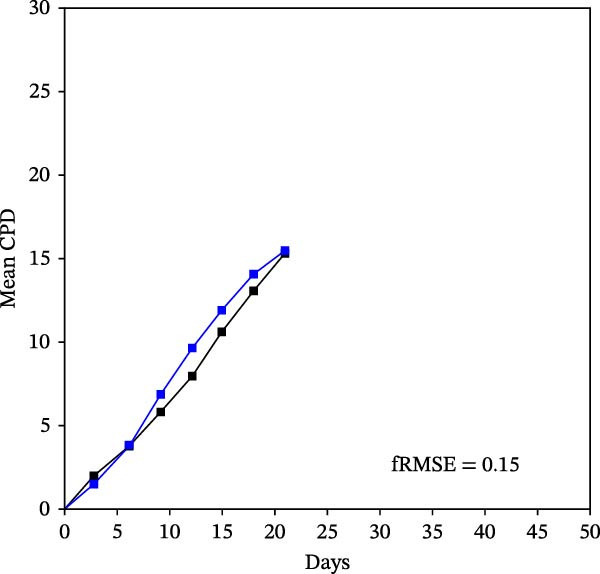
(A)

**Figure:**
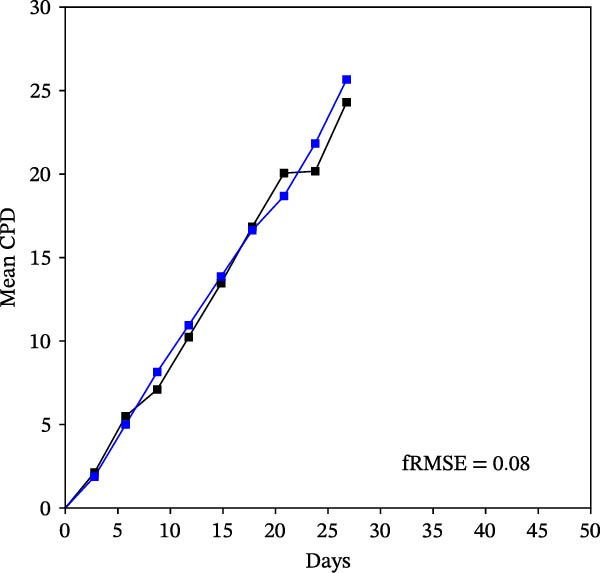
(B)

**Figure:**
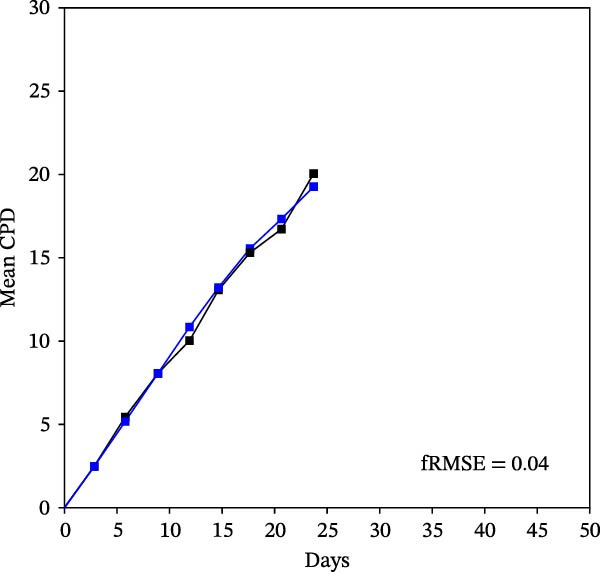
(C)

**Figure:**
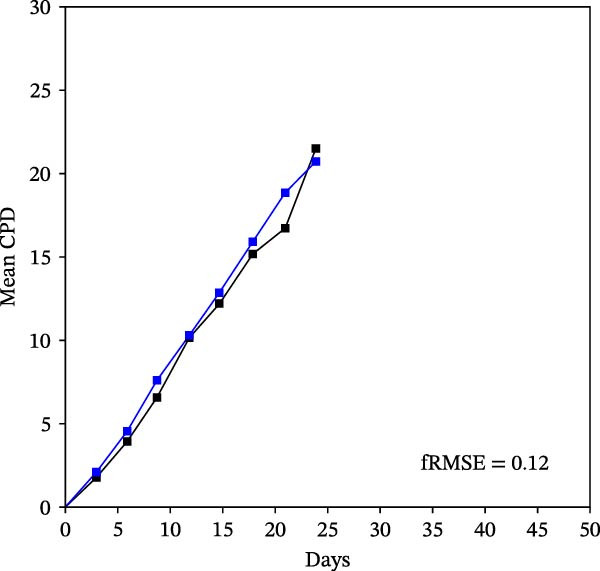
(D)

**Figure:**
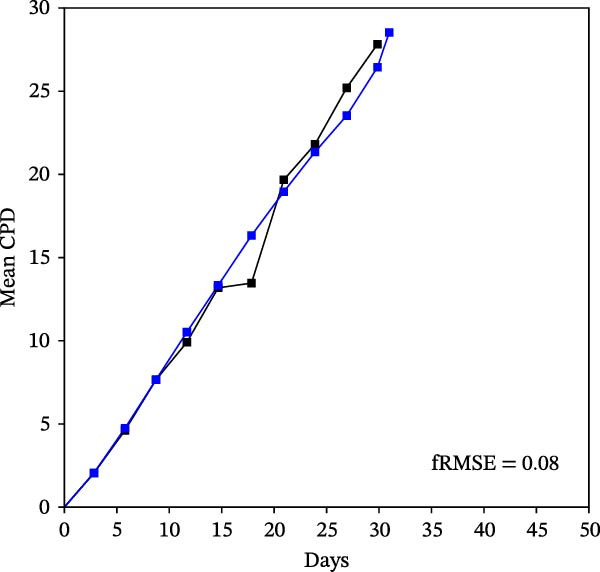
(E)

**Figure:**
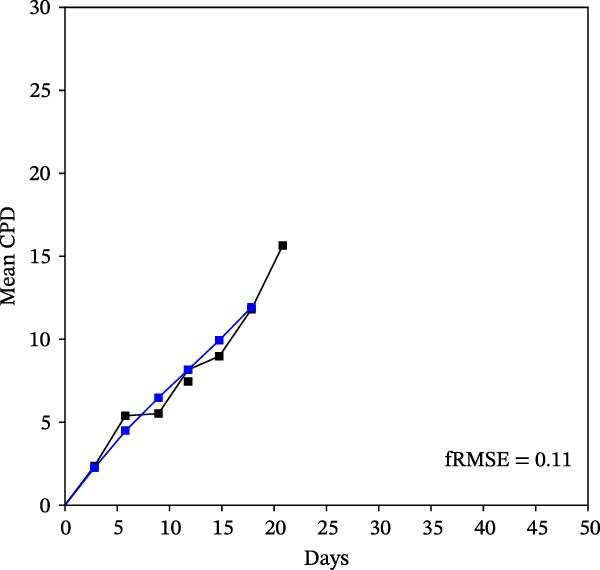
(F)

**Figure:**
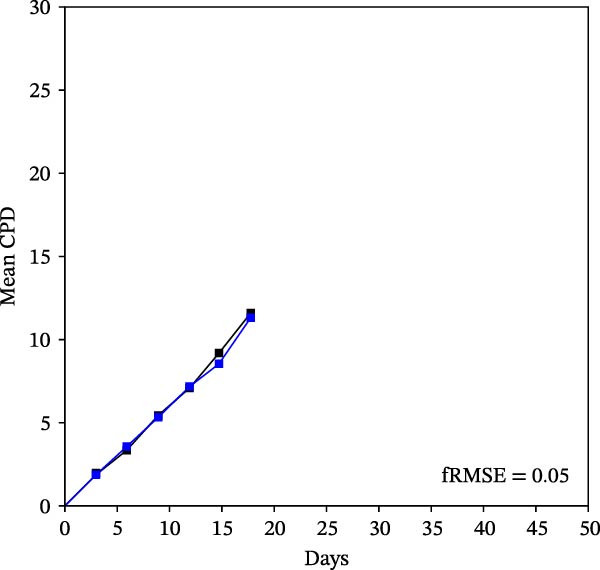
(G)

**Figure:**
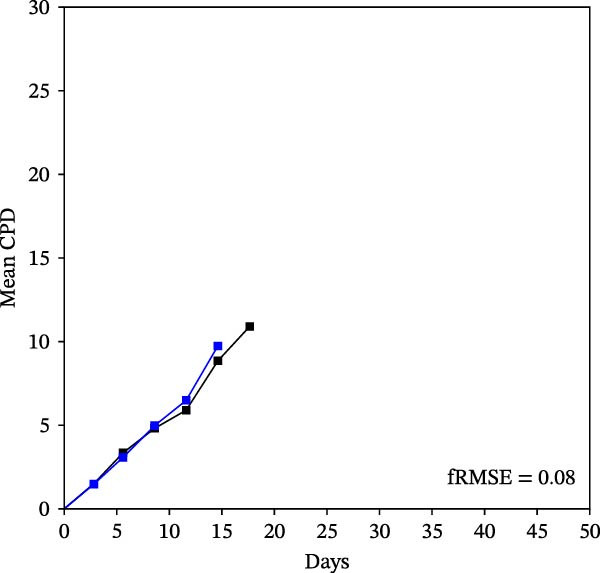
(H)

Figure 3KSC counting TORTOISE Test analyses of cell subtype‐specific cell kinetics profiles during serial cell culture of human alveolar bone‐derived MTC samples. Cell subtype‐specific cell kinetics profiles computed with the initial mean cell kinetics factors (Figure 1 data) used for the respective computer simulations in Figure 2. Shown are the calculated individualized cell kinetics data during passaging for stem cells (blue), transiently dividing committed progenitor cells (red), and terminally arrested cells (green) in terms of their percentage levels. (A, B) Patients 1 and 4, respectively; low SCF stability. (C–E) Patients 3, 8, and 5, respectively; moderate SCF stability. (F–H) Patients 2, 6, and 7, respectively; stable SCF.

**Figure:**
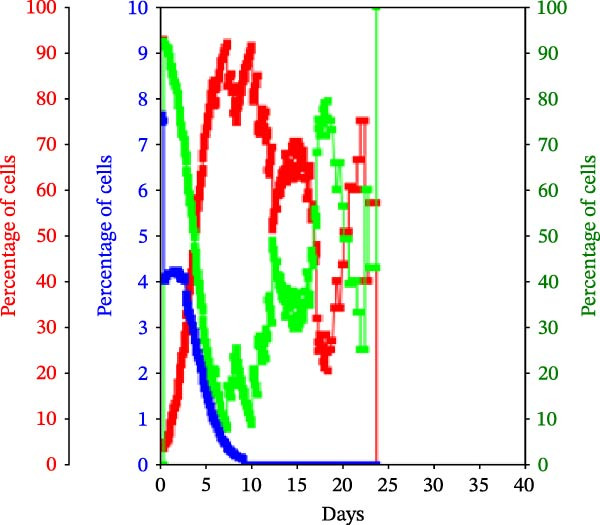
(A)

**Figure:**
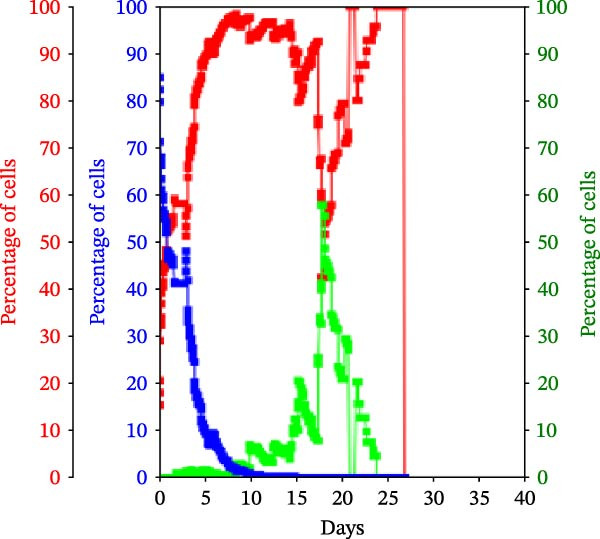
(B)

**Figure:**
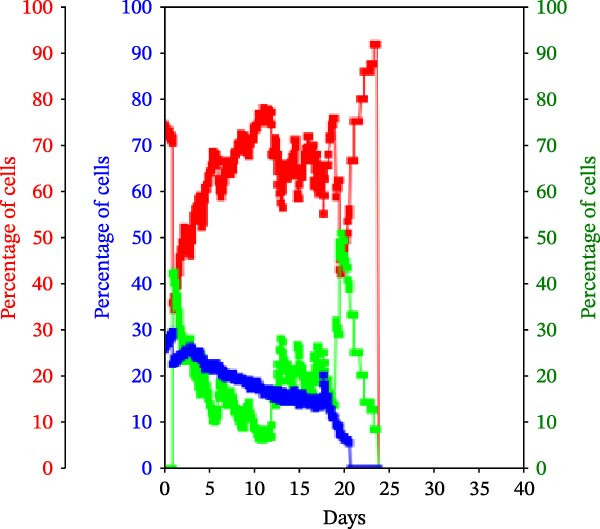
(C)

**Figure:**
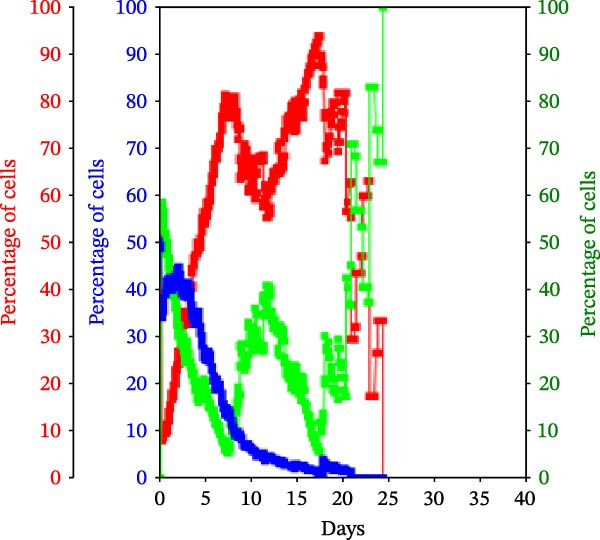
(D)

**Figure:**
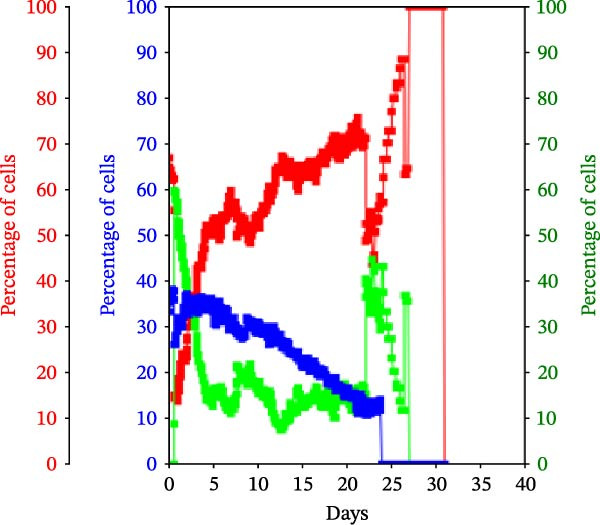
(E)

**Figure:**
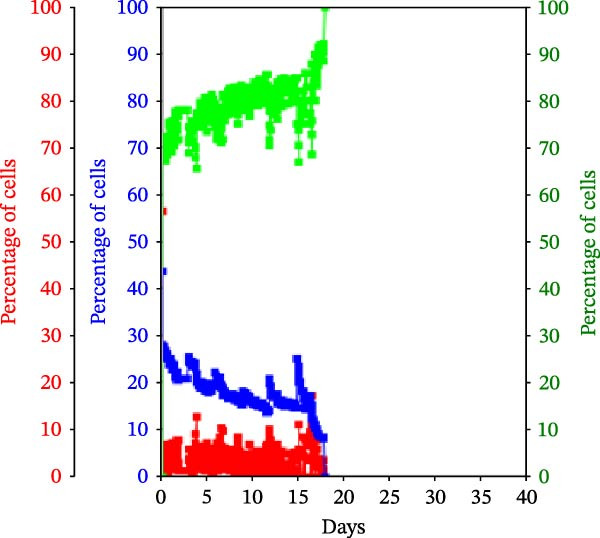
(F)

**Figure:**
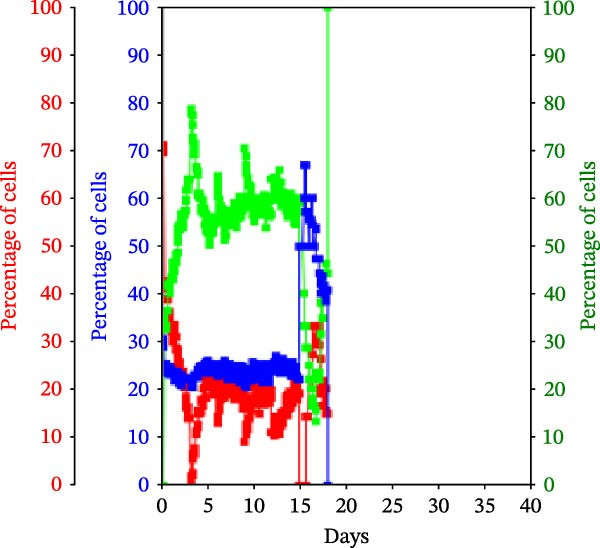
(G)

**Figure:**
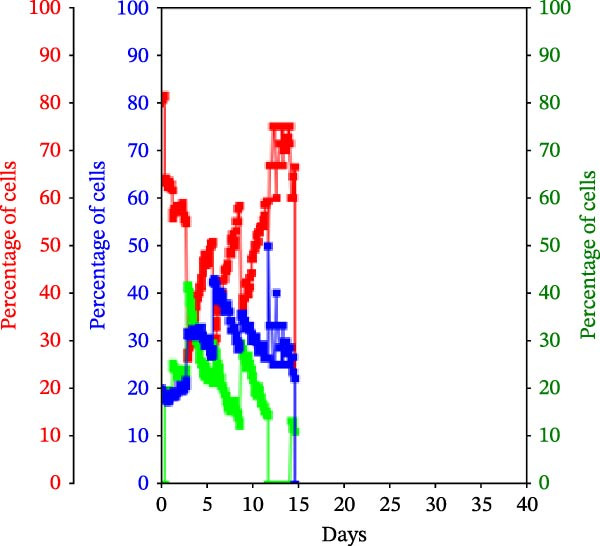
(H)

### 2.3. Statistical Methods

The 95% confidence intervals for TORTOISE Test software mean initial cell kinetics factor determinations were determined using the Student’s two‐tailed *t*‐test with respect to a value of 0.0. The statistics calculations were performed with 2020 GraphPad Prism 9 for macOS software, version 9.0.0. Three‐dimensional cell kinetics factor, cluster graphing was performed with online *Desmos Graphing Calculator* software (https://www.desmos.com/calculator).

## 3. Results

### 3.1. Human Oral Alveolar Bone MTC Preparations Differ in Their SCF Stability

Table 1 provides a summary of the characteristics previously reported for eight independent MTC samples isolated from the alveolar bone of dental patients [14]. Two KSC counting results were reported, the SCF and the SCF half‐life (SCFHL) [12, 13]. KSC counting uses computational simulation to quantify the SCF of tissue cell samples based on a probabilistic model of the cell kinetics program by which stem cells duplicate themselves by symmetric divisions and divide asymmetrically to produce committed progenitor cells and arrested differentiated cells during serial cell culture [11].

**Table 1:** Characteristics of dental patient alveolar bone‐derived MTC samples^a^.

Stability category	Patient #	Sex	Age	SCF^b^	SCFHL^c^
Low stability	1	F	90	0.07	1.61 CPD^d^
	4	F	62	0.77	1.62 CPD
Moderate stability	3	F	78	0.26	4.11 CPD
	8	M	43	0.56	6.05 CPD
	5	M	61	0.37	9.15 CPD
Stable	2	M	56	0.30	NA
	6	F	49	0.22	NA
	7	F	25	0.30	NA

^a^From reference [14].

^b^SCF, stem cell fraction.

^c^SCFHL, SCF half‐life.

^d^CPD, cumulative population doublings.

^#^Patient number.

The KSC counting TORTOISE Test software uses an input of mean CPD data and mean DCF data from serial cell cultures to compute the initial SCF of tissue cell samples and the SCF at any passage during subsequent serial culture. The previously defined donor variation in the initial SCF is evident from the Table 1 data, with MTC sample SCFs ranging from 0.07 to 0.77.

The KSC counting RABBIT Count software relates experimental mean CPD data to the corresponding mean SCF determinations during serial culture to define CPD algorithms [12, 13]. CPD algorithms are first‐order exponential decay equations that can be modeled to estimate the SCFHL of MTCs during serial culture. The SCFHL is the number of CPDs required to achieve a 50% decrease from the initial SCF. It is a measure of the stability of the SCF during serial culture expansion and the first quantitative parameter for declines in stem cell number during expansion culture.

As shown in Table 1, the eight patient MTC samples can be grouped into three distinct categories based on their SCFHL values. Low stability samples had SCFHL values equivalent to 1.6 CPDs, consistent with very rapid losses of MSCs. The SCFHLs of samples with moderate stability ranged from 4 to 9 CPDs. Of particular interest were stable samples, which showed no significant decrease in the SCF with serial culture.

### 3.2. Human Alveolar Bone‐Derived MTC Preparations With More Stable SCFs Have Smaller Stem Cell Turnover Units

In addition to an MTC sample’s initial SCF, the KSC counting TORTOISE Test software estimates a number of other informative cell subtype‐specific cell kinetics parameters. These are diagramed in Figure 1. Together they define the model of cell renewal by tissue stem cells that is the underlying biological basis for the KSC counting method [11]. The model is based on in vitro cell culture evidence that, during serial cell culture, tissue stem cells primarily divide asymmetrically to maintain their own phenotypic state while producing transiently dividing committed progenitor cells [25–27]. The progenitor cell division lineages end with the production of terminally arrested cells. Although the degree of lineage differentiation is limited, variable, and conditional in cell culture, the terminal arrest itself is invariant for normal human tissue cells [25, 28]. At a generally lower frequency, tissue stem cells can divide symmetrically in culture as well, which results in their self‐duplication (Figure 1, symmetric stem cell divisions).

The KSC counting SQS for the analyses of all eight donor MTC samples met the established criterion of being ≤0.5. This requirement ensures a high degree of confidence in the ability of the derived mean initial cell kinetics factors to simulate the experimental CPD data from the serial cultures of each sample [11, 14]. The simulation examples shown in Figure 2 provide an additional quantitative measure of how well the defined mean initial cell kinetics factors are able to simulate their respective experimental mean CPD data when used in TORTOISE Test software computations with the underlying stem cell kinetics model.

Examination of the initial cell kinetics factors of the different MTC samples provided insights into the likely cell kinetics basis for the observed differences in SCF stability. The differences in the rates of MSC production and death were not consistent with these factors being solely responsible. Rates of asymmetric stem cell divisions (i.e., cell cycle times) are not applicable for this analysis, because these divisions do not increase or decrease stem cell number. In contrast, both the frequency and cell cycle time of symmetric stem cell divisions are important determinants of MSC number and fraction.

One of the two MTC samples with low SCF stability had a significantly low frequency of symmetric stem cell divisions (Figure 1A, Patient 1 sample). This difference is predicted to significantly lower the rate of MSC production and is a likely contributor to the low SCF stability. However, the second MTC sample with low stability had a symmetric division frequency well within the range of moderate stability and stable SCF samples. It had a mean symmetric division frequency of 58%, compared to frequencies ranging from 37% to 75% for the more stable samples (Compare Figure 1B to Figure 1C–H). However, it is noteworthy that this sample had the longest mean cell cycle time for symmetric stem cell divisions (25h; Figure 1B), which would contribute to a faster decrease in the SCF if other cell kinetic factors that act to reduce stem cell number were equivalent among the MTC samples.

One such cell kinetic factor for consideration is the rate of stem cell death. However, the rates of stem cell death for the two MTC samples did not provide an explanation. Their rates were either significantly lower or statistically equivalent to the death rates of stem cells in the MTC cultures with moderately stable or stable SCFs. This difference is predicted to increase SCF stability, not lower it.

The initial cell kinetics factors for MTC samples with low SCF stability differed in two other significant respects from the samples with greater stability. The greater distinction was their significantly larger stem cell turnover units. A stem cell turnover unit is the number of cells that constitute the complete committed cell lineage produced by a tissue‐renewing stem cell, including the responsible asymmetrically dividing stem cell [11, 29]. The size, or number of cells in a stem cell turnover unit, is defined by the TDN [11, 29]. The TDN is the number of generations of exponential cell divisions by cells produced from a stem cell division before a final lineage generation undergoes terminal division arrest. The cell turnover unit size equals 2^TDN + 1^ [11, 29].

The two patient MTC samples with the lowest SCF stability had respective mean TDNs of 14 and 16, corresponding to cell turnover unit sizes of 3.3 × 10^4^and 1.3 × 10^5^ cells (Figure 1A,B). The production of this number of progenitor cells and arrested cells for every asymmetric MSC division is predicted to cause a rapid decline in the SCF due to the dilution of MSCs among their progeny cells. Among the MTC samples with greater SCF stability, the greatest mean TDN was only 7, corresponding to a stem cell turnover size of 256 cells (Figure 1D). The TDN values of all the other samples with more stable SCFs were significantly smaller; and the samples showing completely stable SCFs had the smallest stem cell turnover units (Compare Figure 1F–H to Figure 1C–E).

The second distinction of MTC samples with low SCF stability was their lower progenitor cell death rate. On a per‐generation basis, the mean death rate of cells with greater SCF stability ranged from 14% to 26% (Figure 1C–H). In contrast, the mean rate for death estimated for progenitor cells in MTC cultures with low SCF stability ranged from 4% to 11%, and the latter value was not significantly different than 0% (Figure 1A,B). The greater viability of progenitors is another factor that will increase the rate of dilution of MSCs by the cells in their larger cell turnover units. Higher rates of progenitor cell death reduce the rate of stem cell dilution by reducing the effective size of smaller cell turnover units further. In this regard, it is noteworthy that the three MTC samples with stable SCFs had the numerically highest rates of death of terminally arrested cells as well. This difference would also serve to reduce the rate of cell dilution of MSCs and promote stabilization of the SCF with serial expansion culture.

### 3.3. Computational Visualization the Cell Dilution Features of SCF Stability During Serial Expansion Culture

Using the input of the mean initial cell kinetics factors (Figure 1), the KSC counting TORTOISE Test software can compute the respective changes in the fractions of stem cells, progenitor cells, and arrested cells during serial cell culture of tissue cell preparations [11–13]. Examination of such cell subtype‐specific profiles reveals the cell culture dynamics that result from differences in stem cell turnover size and other determinants of SCF stability. As shown in Figure 3, these profiles are highly similar for MTC samples in the same SCF stability group.

The low stability samples both show the most rapid rates of production of progenitor cells to the highest levels (Figure 3A,B, red traces), with arrested cells accumulating to higher fractions later in passage (Figure 3A,B, green traces) compared to the MTC samples with greater SCF stability. These profiles are indicative of the large stem cell turnover units predicted to operate in these cultures. Even though the Patient 1 MTC sample initially has a low SCF due to a high fraction of arrested cells (Figure 3A, green trace), both the initial arrested cells and stem cells (Figure 3A, blue trace) are rapidly decreased in fraction due to dilution by the rapid production of large numbers of progenitor cells in the early cell culture passages.

In contrast, the MTC samples with moderate SCF stability show slower rates of increase in the progenitor cell fraction to lower levels on average (Figure 3C–E, red traces); and arrested cells reach their maximum level at earlier passages (Figure 3C–E, green traces). These cell fraction dynamics are indicative of the smaller stem cell turnover unit size of these cultures and their higher rates of progenitor cell death.

The cell subtype‐specific profiles of the MTC samples with stable SCFs were even more distinctive. Beyond having a stable SCF, the progenitor cell fractions and the arrested cell fractions were also relatively constant during serial culture. Of particular note, arrested cells either maintained a higher fraction than progenitor cells (Figure 3F,G, green traces) or were similar in number to them (Figure 3H, green trace). This feature is predicted by the very small cell turnover units defined for MTC samples with a stable SCF.

## 4. Discussion

Before the development of the KSC counting method, previous reports proposed that an important factor limiting the expansion of tissue stem cells in culture was their dilution among their lineage‐committed progeny cells as a result of their own asymmetric self‐renewal division [10, 11, 25–27]. The earliest results from KSC counting analyses with a variety of different tissue stem cell types supported this proposal and its corollary that suppression of the asymmetric cell kinetics (SACK) of tissue stem cells could be an effective strategy for increasing their production in cell culture [11–13]. In the specific case of MSCs, earlier studies have provided a particularly compelling demonstration of the impact of serial culture on MSC stem cell maintenance. These studies show that whereas intact umbilical cord pieces (UCPs) continued to produce mesenchymal cells for several months in culture, cultures made of the mesenchymal cells that grew out of the same UCPs ceased division after only several passages in culture [30]. This observed distinction was attributed to the retention of MSCs in UCP niches, where they are not diluted by their transiently dividing progeny as they are during serial passaging in culture after their outgrowth or dissociation.

**Figure 4:**
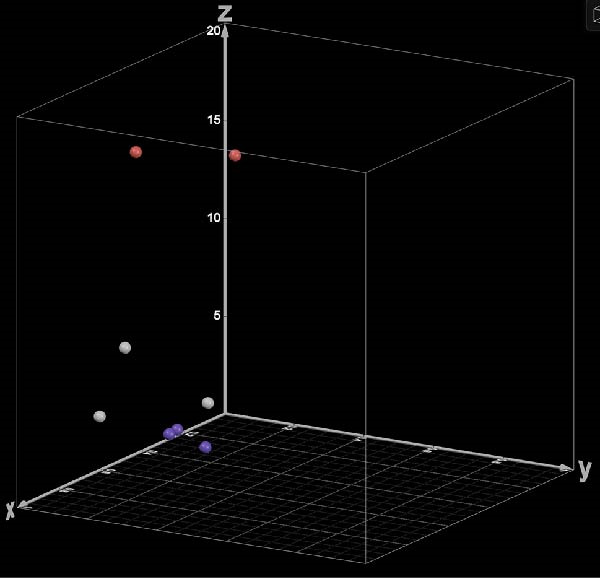
A 3‐dimensional clustering analysis of eight patient alveolar bone‐derived MTC preparations based on three initial mean cell kinetics factors determined by KSC counting analyses. Each datapoint is plotted for the RS value (the rate of symmetric divisions by stem cells, *X*‐axis), the stem cell death rate value (*Y*‐axis), and the TDN value (stem cell turnover division number, *Z*‐axis) for each respective patient MTC preparation. The same data as reported in Figure 1 were used. Red datapoints, patient MTC preparations with low SCF stability; gray datapoints, patient MTC preparations with moderate SCF stability; purple datapoints, patient MTC preparations with stable SCF.

By deploying KSC counting, the precursor studies for the analyses presented herein were the first to confirm quantitatively that there was significant variation in the SCF of the same types of tissue cell preparations isolated from different human patient donors [14]. In addition, those studies revealed that, independent of the initial SCF, alveolar bone MTC preparations isolated from different patient donors had significant differences in the stability of their SCFs during serial culture. Here, we show, by additional KSC counting computational simulation analyses, that the detected differences in SCF stability are largely attributable to differences in the size of the stem cell turnover units produced by the asymmetric self‐renewal division of the MSCs. As highlighted by a three‐dimensional clustering analysis performed with the main three interacting cell kinetics factors that determine the SCF (i.e., RS, the stem cell death rate, and TDN), only TDN provides a clear delineation of the three defined levels of SCF stability during expansion culture (Figure 4). This finding provides a further confirmation of the original hypothesis that a major factor limiting the expansion of tissue stem cells in *in vitro* cell culture is the continuation of their inherent asymmetric self‐renewal division kinetics.

One well‐known feature of serial cell culture that could account for higher SCF stability is clonal evolution of immortalized stem cells, which no longer divide asymmetrically. The presence of stable immortalized stem cell clones in the initial cell isolations or emerging early in the serial cultures could manifest as stable stem cells with small turnover units within the computational resolution of KSC counting analysis. We do not consider this a likely explanation for our findings. Whereas such spontaneous immortalization is common for rodent tissue cells, it is quite rare for normal human cells. In the specific case of MSC strains, though clonal evolution has been observed in lineage‐marking experiments [30], it does not result in the establishment of immortalized cell clones. This property is the basis for the high safety profile of MSCs for transplantation therapies.

An important issue for future evaluation could not be addressed by the present study. Presently it is unclear whether the differences in SCF stability are intrinsic properties of the MTC preparations, reflecting in vivo differences in the donors; or instead the result of extrinsic differences in aspects of their isolation and/or in vitro cell culture. However, all the evaluated MTC preparations were isolated, processed, and cryopreserved by the same procedure; and for the subsequent KSC counting serial culture analyses, they were passaged in the same type of culture medium with the same cell transfer schedule [14]. Recently, we have shown that the serial culture procedures and KSC counting methods are sufficiently repeatable and robust to provide precise SCF determinations even when performed by different investigators, in different laboratories, using different cell counting instruments [31].

Our previous studies implicated fetal bovine serum (FBS) as a possible effector of adipose‐derived MSC stability during expansion culture [12]. Therefore, after completing the studies reported here, we attempted to evaluate whether the observed differences in the stability of the SCF of alveolar bone‐derived MSCs were related to differences in the serum lots used. The many constituents of FBS, which include growth factors and small molecules that influence cell kinetics, are known to be highly variable [32]. Differences in the overall cell proliferation of MTC cultures have been associated with differences in FBS lots [19, 22]. Although some serum lots were not recorded in these initial studies, logs for the dates of the serial culture analyses clearly indicate that patient MTC samples with rapidly declining SCFs and with stable SCFs were performed in parallel. The following MTC samples were serially cultured in parallel: Patient 2 (stable) and Patient 4 (low stability); Patient 6 (stable) and Patient 8 (moderate stability); Patient 1 (low stability) and Patient 3 (moderate stability); Patient 5 (moderate stability) and Patient 7 (stable). This documentation makes it highly unlikely that different lots of serum account for the observed differences in MSC stability during expansion culture.

**Figure 5:**
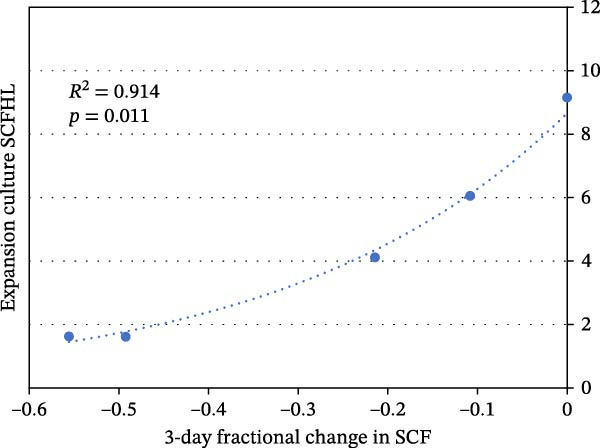
Relationship between early changes in the SCF of human alveolar bone MTC preparations and their SCFHL during long‐term expansion culture. Reported data [14] for the five patient donor cell preparations with low SCF stability or moderate SCF stability were used for the evaluation. The SCFHLs of these donor cell preparations were plotted against the respective differences between their SCFs at time = 0 h and after the initial 3 days of culture. The resulting data were fit to an exponential equation (dotted line). *R* ^2^, Pearson correlation coefficient for the compared values.

Future studies that evaluate MTC preparations derived and quantified with a documented single FBS lot or defined growth factor supplements will resolve this issue definitively. Intrinsic differences, extrinsic growth factor supplement differences, or both might be responsible. If intrinsic differences are at cause, it will motivate further studies to understand their in vivo basis. In this scenario, KSC counting can be used to identify those isolated MTC preparations that have the greater potential for production of MSCs for applications in research and stem cell medicine. A first indication of this beneficial capability is shown in Figure 5. For donor cell preparations with low or moderate SCF stability, the degree of initial decline in their SCF after only 3 days of culture was well correlated with their SCFHL during subsequent expansion culture (*R* ^2^ = 0.914; *p* = 0.011). Such an early indicator would prevent expending time and resources on donor cell preparations that will not be effective for tissue stem cell production.

If extrinsic culture factors like serum lot prove to be major determinants of SCF stability, this knowledge will motivate studies to identify the critical serum components. KSC counting can be used as an assay tool to identify the responsible factors that maintain high rates of symmetric stem cell divisions while reducing the size of asymmetric stem cell turnover units. In any case, whether the cause proves to be intrinsic, extrinsic, or a combination of such factors, the present study further supports the SACK concept of devising new methods to reduce the asymmetric production of progeny cells as an effective strategy for improving biomanufacturing processes for therapeutic tissue stem cells.

KSC counting is a new tool for optimizing the production of tissue stem cells that can be used for research, drug evaluations, and transplantation therapies. In this report, we show how it can be used to develop important actionable clues to differences in the cell production rates of different donor cell preparations. KSC counting can also be used to evaluate the effects of culture medium supplements and conditions on stem cell symmetric division rate, stem cell viability, stem cell turnover unit size, which are independent and interdependent factors that determine overall stem cell production rate [12]. Implementing KSC counting into tissue stem cell production processes promises future identification of more effective production methods that are needed to increase progress in stem cell science and medicine.

## 5. Conclusions

KSC counting computational simulation analyses were performed to investigate how specific cell kinetics factors related to previously reported differences in the stability of the SCFs of MTCs—isolated from the alveolar bone of dental patients—during culture expansion. Characteristically, patient donor samples displaying greater SCF stability had smaller stem cell turnover units, which are composed of stem cell‐descendant committed progenitor cells and arrested differentiated cells. Smaller stem cell turnover units are predicted to cause less dilution of asymmetrically self‐renewing stem cells during tissue cell culture expansions. This new insight from KSC counting analyses suggests more effective strategies for increasing the effectiveness of tissue stem cell biomanufacturing processes.

## Author Contributions

**James L. Sherley**: conception and design, financial support, administrative support, assembly of data, data analysis and interpretation, manuscript writing. **Chen Cao**: conception and design, provision of study materials, collection and assembly of data, data analysis and interpretation. **Celia Sommer**: conception and design, provision of study materials, collection and assembly of data. **Alex Dahlkemper**: provision of study materials, collection and assembly of data. **James Sugai**: provision of study materials, collection and assembly of data. **Hitesh Chopra**: assembly of data, data analysis and interpretation, manuscript writing. **Darnell Kaigler**: conception and design, financial support, administrative support, provision of study materials, data analysis and interpretation, manuscript writing.

## Funding

The study was funded by the National Heart, Lung, and Blood Institute (Grants 1R43HL154900‐01 and 2R44HL154900‐02A1) and the National Institute of Dental and Craniofacial Research (Grants R56DE21410 and R01DE028657).

## Disclosure

All authors reviewed and approved the final manuscript. The funders had no role in the design of the study; in the collection, analyses, or interpretation of data; in the writing of the manuscript; or in the decision to publish the results. The content is solely the responsibility of the authors and does not necessarily represent the official views of the National Institutes of Health.

## Ethics Statement

The MTC populations obtained for the precursor study for this report were derived from alveolar bone samples isolated in accordance with the University of Michigan School of Dentistry Institutional Review Board under approved protocol aBMSCs, IRB# #HUM00034368.

## Consent

Written informed consent was obtained from the patients, who donated the cells for use in the precursor study, at the School of Dentistry, University of Michigan.

## Conflicts of Interest

Asymmetrex LLC is a for‐profit company that markets KSC counting as a commercial service. James L. Sherley is the President and Chief Executive Officer of the company. The other authors declare no conflicts of interest.

## Data Availability

All primary CPD data and DCF data are deposited for free access in Science Data Bank [24].
